# Role for Physical Fitness in the Association between Age and Cognitive Function in Older Adults: A Mediation Analysis of the SABE Colombia Study

**DOI:** 10.3390/ijerph18020751

**Published:** 2021-01-17

**Authors:** Miguel Ángel Pérez-Sousa, Jesús del Pozo-Cruz, Pedro R. Olivares, Carlos A. Cano-Gutiérrez, Mikel Izquierdo, Robinson Ramírez-Vélez

**Affiliations:** 1 Faculty of Sport Sciences, University of Extremadura, 10003 Cáceres, Spain; perezsousa@gmail.com; 2 Epidemiology of Physical Activity and Fitness Across Lifespan Research Group, University of Seville, 41092 Seville, Spain; jpozo2@us.es; 3 Department of Physical Education and Sport, University of Seville, 41092 Seville, Spain; 4 Faculty of Education, Psychology and Sport Sciences, University of Huelva, Avenida de las Fuerzas Armadas s/n 21007, 21007 Huelva, Spain; 5 Instituto de Actividad Fisica y Salud, Universidad Autonoma de Chile, 1670 Talca, Chile; 6 Hospital Universitario San Ignacio–Aging Institute, Pontificia Universidad Javeriana, Bogotá 110111, Colombia; ccano@javeriana.edu.co; 7 Navarrabiomed-Universidad Pública de Navarra (UPNA), Complejo Hospitalario de Navarra (CHN), Instituto de Investigación Sanitaria de Navarra (IdiSNA), Pamplona, 31008 Navarra, Spain; mikel.izquierdo@gmail.com (M.I.); robin640@hotmail.com (R.R.-V.); 8 CIBER of Frailty and Healthy Aging (CIBERFES), Instituto de Salud Carlos III, Pamplona, 28029 Navarra, Spain

**Keywords:** aging, physical function, cognitive status

## Abstract

*Objectives.* We investigated the association between physical fitness and cognitive status. Further, we examined whether physical fitness mediates the association between cognitive functioning and aging. *Design.* Cross-sectional study. *Setting.* Urban and rural Colombian older adults. *Methods.* 4416 participants from the SABE study were included in the current analysis. Physical fitness was assessed with the handgrip test and the usual gait speed test. Cognitive status was evaluated through the Folstein Mini-Mental State Examination. A parallel mediation path was used to test the possible mediator role of physical fitness between aging and cognitive functioning. *Results.* Older adults with lower handgrip strength (HGS) were more likely to have mild-cognitive status than older adults with healthy HGS (OR = 1.53, 95% CI = 1.15; 2.02). In addition, older adults with a slower gait speed were more likely to have mild cognitive impairment (OR = 2.05, 95% CI = 1.54; 2.78). Age had an inverse relationship with cognitive function (β = −0.110, 95% CI = −0.130; −0.100) and it was also inversely associated with HGS (β = −0.003, 95% CI = −0.005; −0.002) and gait speed (β = −0.010, 95% CI = −0.011; −0.009). The indirect effects, which indicate that the effect of age on cognitive function is transmitted through mediators, showed that both gait speed (β = −0.028, 95% CI = −0.036; −0.020) and HGS (β = −0.014, 95% CI = −0.024; −0.005) were independent mediators of the detrimental effect of aging on cognitive function. *Conclusions.* Physical fitness mediates the effects of aging on cognitive functioning. Our findings suggest that physical activity can be a key factor to prevent cognitive deterioration during aging process.

## 1. Introduction

The Latin-American population is aging fast, and it has been projected that by 2050 the number of people older than 65 will double [1]. Aging is associated with several non-communicable diseases, including mobility disability [2] and cognitive decline [3]. In Colombia (South America), the prevalence of mild cognitive impairment is increasing and currently stands at 5.6% [4]. Mild cognitive impairment in older adults leads to deficits in activities of daily living and quality of life [5], and its progression to more serious cognitive problems (e.g., dementia) is associated with early mortality [6]. Accordingly, identifying risk factors that can help mitigate or delay the appearance of cognitive impairment is a key challenge for health care systems.

Maintenance of physical fitness through the adoption of a physically active lifestyle is known to promote healthy aging [7]. Physical fitness can be defined as a set of measurable attributes that people achieve through physical activity and that are associated with physical and mental well-being [8]. In older adults, physical fitness is typically assessed through specific tests, including handgrip, balance, and gait speed [8], which provide an overview of motor and muscle strength competence.

Physical fitness can decrease dramatically with age, and numerous studies have highlighted the deterioration in muscular strength, balance, gait speed, mobility, and cardiorespiratory performance in men and women aged >60 years of age [9,10,11]. A better physical fitness status is associated with better health and quality of life [12,13].

Accumulating evidence indicates that a relationship between physical fitness and cognition exists and that a decline in physical performance precedes the deterioration of cognitive ability. For example, A recent study based on the UK Biobank study (2007–2010) of 476,559 participants highlighted that muscle strength measured by a handgrip test was positively and prospectively associated with memory and processing speed [14]. A similar study with 6874 older adults found that physical activity level and lower-limb muscle strength predicted a lower cognitive function [5]. Conversely, other studies have found the opposite: cognitive decline leads to a lower physical performance. For example, in a longitudinal study of over 3500 participants from The Netherlands, Stijntjes et al. [15] found that a poorer executive function was associated with a steeper decline in gait speed in people aged 55–90 years. Likewise, in the Baltimore Longitudinal Study of Aging (412 participants aged ≥60 years), Tian et al. [16] found that the relationship between usual gait speed and executive function was unidirectional, such as a slower walking speed predicted future declines in executive function and memory but not vice versa. Finally, a prospective study of 2876 well-functioning older adults (70–79 years) from the US found that early declines in gait speed predicted a decline in orientation, attention, calculation, language and short-term memory, but the association between early declines in cognition and later declines in gait speed was weaker [17]. Thus, the empirical evidence connecting the two phenomena is rather inconclusive.

Regarding the potential beneficial relationship between exercise training and cognitive functioning, Baker et al. used a rigorously controlled methodology to examine aerobic exercise’s effects on cognition in 33 adults (mean age 70 years) with mild cognitive impairment. The authors of the former study found an improvement of executive function through aerobic exercise in older women but not men [18]. Likewise, Zhihui et al. [19] reported in a systematic review of randomized clinical trials on the beneficial effects of resistance training on cognitive function in older adults. Exercise-induced changes in cognitive status could be explained by modifications to brain characteristics and functioning, as exercise induces cognitive plasticity [20], improves cerebral perfusion [21] and cerebrovascular reactivity [22], and reverses hippocampal volume loss, thereby improving memory [23].

While it is clear that aging is associated with cognitive decline and changes to cognitive functioning, to our knowledge, no studies have examined the potential mediating role of physical fitness measured through gait speed and HGS on the association between aging and cognitive decline. Consequently, the present study was designed to evaluate the association between physical fitness and cognitive status and to examine whether physical fitness mediates the decline in cognitive functioning associated with aging.

## 2. Methods

### 2.1. Design, Setting and Participants

We analyzed data from the “Estudio Nacional de **Sa**lud, **B**ienestar y **E**nvejecimiento” (SABE) Colombia survey. SABE is a nationwide, population-based, cross-sectional study that was conducted in 2015 by the Epidemiological Office of the Ministry of Health and Social Protection of Colombia (https://www.minsalud.gov.co/). Data were obtained using a probabilistic sampling scheme by clusters (housing segments) with block stratification. The estimated sample size was 24,553 adults aged 60 years and above, assuming an 80% response; the target sample was 30,691 individuals [24]. Nonetheless, because of variations in the application of strategies to achieve a response rate of 70% across regions and civil settings (i.e., urban/rural distributions), the final sample size included 23,694 from 244 municipalities across all departments. More details about the study design and protocol can be consulted in the research published by Gomez et al. [24].

**Figure 1:**
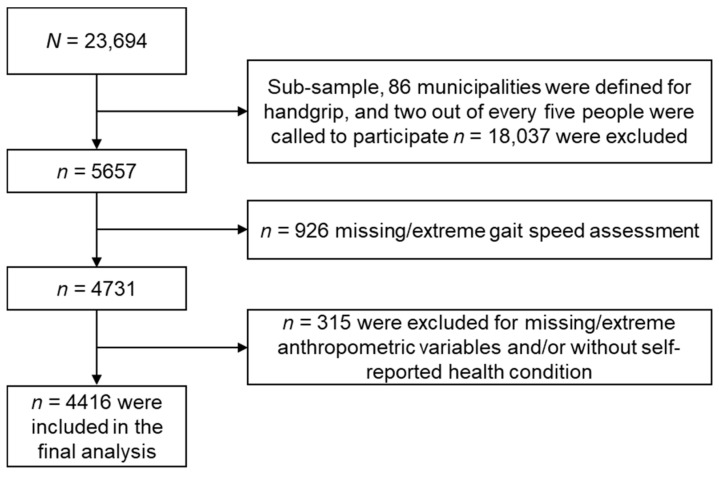
The flow chart shows the study sample selection from the Colombian Health and Wellbeing and Aging Survey (SABE) 2015. All analyses presented here were based on 4416 surveyed participants, each with complete HGS and long-term condition data.

For this subsample analysis, 86 municipalities were selected, including the 4 large cities (Bogota, Cali, Medellin and Barranquilla), for the application of functionality tests and muscle strength assessment, obtaining a sample of 5657 people 60 years of age or older. Of these participants, 4146 subjects were selected for this study. We excluded those with missing data of HGS, gait speed tests and/or anthropometric variables and/or without self-reported health condition (see Figure 1). All participants (or their proxy respondent) provided written informed consent for their data to be used in the study.

### 2.2. Data Collection

The health survey included medical history examination, physical fitness examination and questionnaires on health disorders history, lifestyle data and anthropometric variables. Physical tests were performed by technical, medical staff following the standardized protocol for the SABE study [24].

#### 2.2.1. Sociodemographic, Health Disorders History and Lifestyle Data

Participants were asked about sociodemographic factors, including ethnic group (indigenous, black “Mulatto” or Afro-Colombian, white, others and non-ethnic), living area (rural or urban), and socioeconomic status (SES): level I–II: low; level II–III: middle; and level V–VI: high. Three lifestyle variables were included in the survey. Alcohol consumption was assessed using the question: “In the last three months, on average, how many days of the week have you had alcoholic beverages?” Responses were divided into four categories: (1) no alcohol consumption, (2) 1–2 glasses per day, (3) 3–5 glasses per day, (4) more than 5 glasses per day. The variable was then dichotomized by grouping categories 2–4 as alcohol consumption, and category 1 as no alcohol consumption. 

Smoking was assessed by asking individuals if they were currently smoking or had ever smoked. Answers were divided into four categories: (1) never smoked, (2) former smoker, (3) smokes less than 5 cigarettes per day, (4) smokes more than 5 cigarettes per day. This variable was also dichotomized by grouping categories 1 and 2 as not smokers and 3 and 4 as smokers. The following questions were used to assessed a “proxy” for physical activity: (1) “Have you regularly exercised, such as jogging or dancing, or performed rigorous physical activity at least three times a week for the past year?”; (2) “do you walk at least three times a week between nine and 20 blocks (1.6 km) without resting?”; (3) “do you walk at least three times a week eight blocks (0.5 km) without resting?”. Participants were considered physically active if they responded affirmatively to two of the three questions [25]. 

Medical information including multimorbidity, as well as chronic condition adapted from the original SABE study [24], was assessed by asking the participants if they had been medically diagnosed with hypertension, diabetes, chronic obstructive pulmonary disease, CVD (heart attack, angina), stroke, different types of cancer, arthritis, osteoporosis, cholesterol, triglycerides, mental or sensory problems.

#### 2.2.2. Anthropometrics Measurement

Height and body weight were measured by a portable stadiometer (SECA 213, Hamburg, Germany) and an electronic scale (Kendall graduated platform scale). BMI was calculated as weight in kilograms divided by the square of height in meters.

#### 2.2.3. Physical Fitness Tests

HGS was used to measure the muscle force profile of the upper limb. For this, we used the Takei dynamometer (Takei Scientific Instruments Co., Tokyo, Japan). Prior to the assessment, the dynamometer was calibrated to ensure proper usage and accuracy. Subjects were asked to perform the assessment (with the elbow joint in full extension) while standing if possible and were given a practice trial to ensure comprehension of the procedure. The grip tests were performed three times on each hand, alternating hands between each trial, and the mean value was recorded as the final score of the test. Testers ensured a total of 60 s of rest between trials on the same hand. The values were normalized to body weight (relative HGS).

Usual gait speed (meters/second) was measured by 3 m walking. The participants had to walk two times at the usual pace starting from a standing position.

#### 2.2.4. Cognitive Function

Cognitive status was assessed using the revised version of the Folstein Mini-Mental State Examination (MMSE), a validated international scale translated to Spanish [26]. The modified version ranges from 0 to 19, with a higher score representing better cognitive function.

### 2.3. Statistical Analysis

At first, univariate analysis was used to explore extreme values and Kolmogorov Smirnov was used to examine data distribution. Categorical variables are presented using frequencies and percentages, and continuous variables are presented using means and standard deviations. We applied covariance analysis adjusted by sex, age, lifestyle and comorbidities variables to explore the physical fitness differences between cognitive status groups. Unadjusted and adjusted logistic regressions controlling for age, sex, lifestyle and comorbidities were employed to assess the likelihood of having cognitive impairment based on physical fitness level. According to the literature, the covariates included in the adjusted analyses were based on the conceptual model [14,15,16,17]. For this purpose, we used the European Working Group on Sarcopenia in Older People 2 (EWGSOP2) guidelines and criteria to determine sarcopenia from the assessment of gait speed (<0.80 m/s) and HGS (<27 kg in men and <16 kg in women) [27]. 

**Figure 2:**
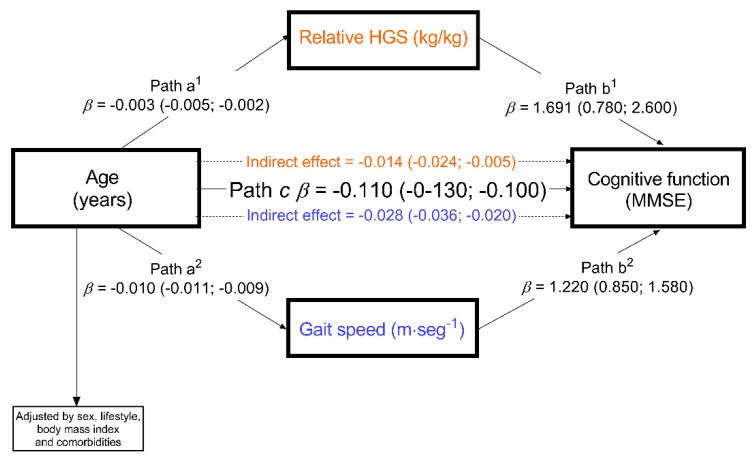
Parallel mediation analysis of aging effects on cognition (MMSE) score through relative HGS (kg) and gait speed (m·seg^−1^), adjusted by sex, lifestyle, body mass index and comorbidities. Number of bootstrap samples = 5000. The indirect effect is statistically significant at the 95% confidence interval (CI) when the CI does not include 0. Betas (β) are reported as the product of simultaneous regression with bootstrap replacement Path a^1^ & a^2^ = association between age and relative HGS and gait speed, respectively; Path b^1^ & b^2^ = association between relative HGS and gait speed with cognitive function; Path c = direct effect; orange and blue associations = indirect effect by relative HGS and gait speed, respectively.

To test for the possible mediator role of physical fitness between aging and cognitive status, we designed a parallel mediation path analysis (see Figure 2). The mediation model indicated through estimation of indirect effects, what physical fitness components (handgrip strength or/and gait speed) were mediators between the detrimental impact of age on cognitive function as assessed by the MMSE test [26]. To perform the analysis, we used the PROCESS macro for SPSS (IBM, Chicago, IL, USA) [28]. The mediation hypothesis was tested using the bias-corrected bootstrap method with 5000 samples to calculate confidence intervals (95%). The point estimate was considered significant when the confidence interval did not cross zero. In addition, we used the test included in the PROCESS macro to compare indirect effects. Significance was set at the *p* ≤ 0.05 level.

## 3. Results

The descriptive characteristics of participants are presented in Table 1. The mean age of participants was 69.5 ± 7.1 years. The distribution by sex in the overall sample was 57.3% for females and 42.7% for males. Of the 4416 participants included in the study, 510 (11.5%) showed mild cognitive impairment. Regarding the distribution across the different SES levels, the majority of participants fit in the lowest SES level (level I–II). The lifestyle outcomes showed that the proportion of individuals drinking alcohol and smoking was low, 13.5% and 11.0%, respectively. A significant proportion of older adults (80.6%) did not accomplish the minimum required daily physical activity “proxy”. Regarding comorbidities, visual problems, high blood pressure and cholesterol had the highest percentage of incidence. There were significant differences between healthy individuals and individuals with cognitive impairment for all variables tested.

Table 2 shows the performance and differences in physical fitness according to cognitive status. Statistically significant differences in HGS relative to body weight were found between older healthy adults and their peers with cognitive impairment after adjusting for sex, age, lifestyle characteristics and comorbidities. The best performance was 0.34 kg/kg *versus* 0.30 kg/kg in healthy older adults *versus* peers with poor cognitive functioning. Similar results were observed for gait speed, with older adults without cognitive impairment showing better functioning than those with poor cognition. Analysis of covariance revealed statistically significant differences (*p* < 0.001) after adjusting for sex, age, lifestyle outcomes and comorbidities.

Table 3 shows the associations in odds ratios between low HGS and low gait speed according to the EWGSOP2 cut-off and mild cognitive impairment. Older adults with low HGS were more likely to have mild-cognitive impairment than older adults with healthy muscle strength after adjusting for age, sex, lifestyle characteristics and comorbidities (OR = 1.55, 95% CI = 1.16; 2.03). In addition, older adults with slow gait speed were more likely to have mild cognitive impairment (OR = 2.08, 95% CI = 1.56; 2.80).

As shown in the mediation model (Figure 2), we found that the independent variable (age) had an inverse relationship with cognitive function (β = −0.110, 95% CI = −0.130; −0.100). Age was inversely associated with HGS (β = −0.003, 95% CI = −0.005; −0.002) and gait speed (β = −0.010, 95% CI = −0.011; −0.009). The indirect effects showed that both gait speed (β = −0.028, 95% CI = −0.036; −0.020) and HGS (β = −0.014, 95% CI = −0.024; −0.005) were independent mediators of the detrimental effect of aging on cognitive function.

## 4. Discussion

The present study analyzed a representative sample of Colombian older adults from a National Survey (SABE). We examined the association between low HGS (men <27 kg, women <16 kg) and low gait speed (<0.80 m/s) and cognitive impairment. We also studied the relationship between age and cognitive functioning and whether this relationship was mediated by physical fitness measured through gait speed and HGS. 

This study’s main finding was the mediator role of gait speed and HGS between aging and cognitive impairment using a parallel mediation model. Our findings suggest that the association between age and cognition is mediated by the level of HGS and gait speed. Accordingly, the loss in cognitive function associated with age could depend on the individual level of physical fitness. For example, older adults with poorer HGS or/and gait speed would show an accelerated loss in cognitive impairment, and the opposite would be seen in peers with a better performance on HGS or/and gait speed. The findings indicate that gait speed and HGS mediate the deterioration of the cognitive status associated with aging, and thus are active components of the aging effects on cognitive status. To the best of our knowledge, this is the first study examining the mediating role of physical fitness in the relationship between age and cognition. Our findings are consistent with the idea that physical fitness contributes to better cognitive functioning [14,29].

The mediator effect of physical fitness could be explained by the benefits that physical exercise has on cognitive health. For example, Xu et al. [30] found an improvement in cerebral perfusion in older women (but not men) after one session per week of resistance training. Strength training also leads to beneficial changes in white matter atrophy and neuroplasticity [31]. The results from two systematic reviews [31,32] revealed that older adults who participated in a resistance training program maintained or improved their neuroplasticity and brain atrophy. The hippocampus is known to shrink in late adulthood, and exercise training has been shown to increase the hippocampal volume, including high-intensity interval training [33] and strength exercise [34]. Similarly, a multicomponent exercise program, including strength, aerobic and balance exercises, was found to reduce whole brain cortical atrophy in older patients with mild cognitive impairment compared with a control group [35], resulting in improved cognitive function. A possible underlying mechanism for these beneficial changes in brain characteristics and functioning is the increase in production and secretion of brain-derived neurotrophic factor [36,37,38].

Another finding showed that Colombian older adults with a poor performance in HGS and gait speed were 1.53 and 2.05 times more likely, respectively, to experience mild cognitive impairment. Our findings are supported by prior research showing that a decline in cognition is led by lower physical fitness performance. For example, it was shown that lower HGS was associated with a poorer performance in memory and processing speed [14]. Likewise, gait speed was shown to predict cognitive impairment and dementia [39]. However, other studies indicate the opposite, with several showing that the relationship between physical fitness and cognition is bidirectional or that cognitive decline precedes poor HGS and gait speed [15,16,17]. From our perspective, the deterioration in cognitive function is preceded by low physical activity and, consequently, by poor physical fitness. This is based on the beneficial effects that exercise has on cognitive health, as shown in previous studies [40,41]. With this in mind, the main objective of the present study was to assess whether physical fitness mediates the inverse relationship between age and cognition.

The present study has several strengths. It is based on a large sample size of older adults within a nationally representative proportion of persons aged ≥60 years. In addition, we used a direct measure of physical fitness, a far more reliable and valid measure than self-report questionnaires. HGS and gait speed also are the two most used measures of physical fitness levels used in older adults [42,43,44]. Moreover, the novelty of examining the mediator role of physical fitness comparing HGS and gait speed should also be considered a strength. Thus, there should be more emphasis on physical fitness in subsequent iterations of the mental health act of Colombia as part of a policy to improve cognitive status across the life course.

Our study does have several limitations. First, the cross-sectional design limits the ability to draw on causal associations. Second, even though we adjusted for potential confounding factors such as sex, lifestyle variables and comorbidities, the population was heterogeneous, especially regarding ethnicity. Some variables were self-reported and are subject to biases. In addition, cognitive status was assessed by a unique self-reported questionnaire, but it is not a clinical diagnosis of cognitive impairment. It would thus be advisable in future studies to combine several questionnaires to avoid bias.

Our finding offers insight into the potential role of fitness on cognitive decline in older adults. Specifically, it is clear that both gait speed and muscle strength must be addressed in future anti-aging programs. Specifically, and following the EWGSOP2 [27] recommendations, the HGS should be above 27 kg for men and 16 kg for women, and gait speed should be >0.80 m/s.

## 5. Conclusions

Physical fitness, measured by gait speed and HGS, mediates the relationship of aging on cognitive functioning in older adults in Colombia and elsewhere in Latin America & the Caribbean. Our findings suggest the need to maintain gait speed and HGS in older adults to avoid cognitive function loss.

## Figures and Tables

**Table 1:** Sample characteristics stratified by cognitive status.

Sociodemographic Characteristics	Overall	No Cognitive Impairment *n* = 3906 (88.5%)	Cognitive Impairment *n* = 510 (11.5%)	*p*-Value
Sex (female), *n* (%)	2531 (57.3)	2224 (56.9)	307 (60.2)	0.001
Sex (male), *n* (%)	1885 (42.7)	1682 (43.1)	203 (39.8)	
Age group, *n* (%)				
60–69	2512 (56.9)	2366 (60.6)	146 (28.6)	0.001
70–79	1431 (32.4)	1234 (31.6)	197 (38.6)	
80+	473 (10.7)	306 (7.8)	167 (32.7)	
Nutritional status, *n* (%)				
Underweight	83 (1.9)	73 (1.9)	10 (2.0)	0.001
Normal weight	1344 (30.4)	1141 (29.2)	203 (39.8)	
Overweight	1809 (41.0)	1628 (41.7)	181 (35.5)	
Obese	1180 (26.7)	1064 (27.2)	116 (22.7)	
Socioeconomic status, *n* (%)				
Level I–II (low)	3371 (76.3)	2934 (75.3)	428 (83.9)	0.001
Level III–IV (medium)	1007 (22.8)	926 (23.7)	81 (15.9)	
Level V–VI (high)	38 (0.8)	37 (0.9)	1 (0.2)	―
Ethnic group, *n* (%)				
Indigenous	267 (6.0)	267 (6.8)	0 (0.0)	―
Black	369 (8.4)	369 (9.4)	0 (0.0)	―
White	1234 (27.9)	1234 (31.6)	0 (0.0)	―
Others	2036 (46.1)	2036 (52.1)	0 (0.0)	―
Non-ethnic	510 (11.5)	0 (0.0)	510 (11.5)	―
Living area, *n* (%)				
Urban	3406 (77.1)	3060 (78.3)	346 (67.8)	0.001
Rural	1010 (22.9)	846 (21.7)	164 (32.2)	
Lifestyle outcomes, *n* (%)				
Alcohol	594 (13.5)	559 (14.3)	35 (6.9)	0.001
Smoking	487 (11.0)	422 (10.8)	65 (12.8)	
Non-physically active	3555 (80.6)	3098 (79.3)	457 (89.8)	
Comorbid chronic diseases, *n* (%)				
HBP	2374 (53.9)	2077 (53.3)	297 (58.3)	0.001
High cholesterol	2159 (49.1)	1930 (49.6)	229 (45.5)	
Diabetes	715 (16.2)	635 (16.3)	80 (15.7)	
Cancer (any type)	210 (4.8)	195 (5.0)	15 (2.9)	
COPD	443 (10.0)	375 (9.6)	68 (13.4)	
CVD	600 (13.6)	520 (13.3)	80 (15.7)	
Stroke	167 (3.8)	138 (3.5)	29 (5.7)	
Arthritis	1192 (27.1)	1075 (27.6)	117 (23.1)	
Osteoporosis	499 (11.4)	446 (11.5)	53 (10.4)	

Categorical variables are reported as numbers and percentages in brackets. Significant between-cognition status differences χ^2^. Comorbidities are reported as “presence/yes”. HBP: high blood pressure; COPD: chronic obstructive pulmonary disease; CVD: cardiovascular disease.

**Table 2:** Physical fitness performance in Colombian older adults according to cognitive status.

Variables	No Cognitive Impairment	Cognitive Impairment	Model 1 *p*-Value	Model 2 *p*-Value	Model 3 *p*-Value
Absolute HGS (kg)	22.08	18.50	<0.001	<0.001	<0.001
Relative HGS (kg/kg)	0.34	0.30	<0.001	<0.001	<0.001
Gait speed (m·s^−1^)	0.77	0.63	<0.001	<0.001	<0.001

Note: Model 1: adjusted by sex and age; Model 2: adjusted by Model 1, ethnicity, urbanicity, socioeconomic status and lifestyle; Model 3: adjusted by Model 2 and comorbidities.

**Table 3:** Physical fitness association with cognitive function in Colombian older adults.

	Model 1			Model 2			Model 3		
	OR	95% CI	*p*-Value	OR	95% CI	*p*-Value	OR	95% CI	*p*-Value
Lower HGS	1.45	(1.11; 1.90)	0.006	1.47	(1.11; 1.93)	0.006	1.55	(1.16; 2.03)	0.002
Lower Gait speed	2.12	(1.61; 2.79)	0.007	2.06	(1.55; 2.72)	<0.001	2.08	(1.56; 2.80)	<0.001

Note: Lower HGS and gait speed are defined according to EWGSOP2 guidelines (<0.80 m/s for gait speed) (<27 kg in men and <16 kg in women for HGS). Model 1: adjusted by sex and age; Model 2: adjusted by Model 1, ethnicity, urbanicity, socioeconomic status and lifestyle; Model 3: adjusted by Model 2 and comorbidities.

## Data Availability

The current study used data from the Ministerio de Salud y la Protección Social de Colombia (https://www.minsalud.gov.co), and legal constraints do not permit public sharing of the data. The Ministerio de Salud y la Protección Social de Colombia, however, is open to all qualified researchers anywhere in the world. Thus, the data used in this communication can be easily and directly accessed by applying through the Ministerio de Salud y la Protección Social de Colombia Management System (https://www.sispro.gov.co/pisis/Pages/pisis-plataformade-integraci%C3%B3n-de-SISPRO.aspx).
